# Multicompartment Darcy Flow Model With Patient‐Specific Parameterization: Effect of Heterogeneity and Anisotropy in Porous Parameters

**DOI:** 10.1002/cnm.70091

**Published:** 2025-09-09

**Authors:** Namshad Thekkethil, Hao Gao, Nicholas A. Hill, Xiaoyu Luo

**Affiliations:** ^1^ School of Mathematics and Statistics, University of Glasgow Glasgow UK

**Keywords:** Darcy flow, multicompartment flow, myocardial perfusion, poroelasticity

## Abstract

Blood perfusion in cardiac tissues involves intricate interactions among vascular networks and tissue mechanics. Perfusion deficit is one of the leading causes of cardiac diseases, and modeling certain cardiac conditions that are clinically infeasible, invasive, or costly can provide valuable supplementary insights to aid clinicians. However, existing homogeneous perfusion models lack the complexity required for patient‐specific simulations. In this study, we develop a computational framework for modeling perfusion using a multicompartment Darcy flow model with heterogeneous anisotropic perfusion that incorporates the nonlinear deformation and compliance of blood vessels with poroelastic parameters derived from realistic vascular data. Through numerical simulations and a comparison of pore pressure results obtained from the proposed model and the Poiseuille flow approach in a benchmark problem, we demonstrate that the heterogeneous anisotropic model outperforms homogeneous models in predicting perfusion, particularly by accurately capturing the spatial heterogeneity of the poroelastic parameters and the permeability transitions from large vessels to microvessels. Additionally, the proposed model successfully simulates patient‐specific conditions, such as vessel blockages, highlighting its potential for personalized medical applications.

## Graphical abstract

Blood vessels in the microvasculature divided into multiple compartments, illustrating flow between compartments within a representative volume element and the pressure distribution predicted by the multicompartment Darcy flow model.

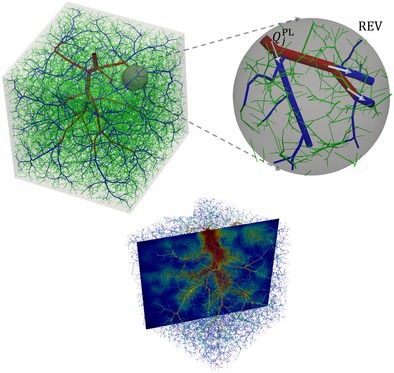

## 1 Introduction

Cardiac diseases rank among the leading causes of death worldwide. Despite advancements in diagnostic techniques, certain cardiac conditions, especially those involving the microvasculature, remain challenging to detect with current non‐invasive methods. Coronary artery disease (CAD), particularly small vessel disease (SVD), presents a significant diagnostic challenge due to the limitations in measuring the dynamics of small and microvessels. This diagnostic gap leaves patients at risk, even post‐treatment, for conditions like angina following percutaneous coronary intervention (PCI) [1]. As a result, supplemental diagnostic methods are needed to enhance our understanding and analysis of SVD.

Computational techniques have emerged as promising tools for obtaining data on small and microvessels using methods such as constrained constructive optimization [2, 3]. Having a detailed coronary vasculature model allows for computational methods to assess blood flow in patient‐specific small and microvessels, capturing the complexity of microcirculatory flow and myocardial mechanics. This is especially important for microcirculation within the myocardium, where conditions like blockages in small or microvessels can significantly affect perfusion, leading to various SVD‐related heart issues.

A poroelastic framework is commonly employed to model myocardial perfusion with coronary blood flow, coupled with ventricular dynamics [4, 5, 6]. Many earlier studies have utilized homogeneous, isotropic poroelastic models, which average discrete vessel data into a simplified micro model [6, 7]. These models assume constant permeability and porosity throughout the tissue, which, although suitable for healthy heart perfusion [7], may not accurately capture the effects of SVD. For example, traditional models using Darcy flow average pressures and flows across pores, making it difficult to differentiate between arterial, capillary, and venous pressures. Moreover, structural features of capillary networks, such as tortuosity and influence on oxygen transport [8], play a critical role in regulating intramyocardial microcirculation [9]. Flow and material properties also vary significantly based on vessel diameter [10], necessitating a more nuanced approach to accurately represent blood flow dynamics in diseased states.

To address this complexity, multicompartment models have been suggested as effective for simulating heterogeneous myocardial blood flow distribution [11]. Some earlier studies adopted two‐porosity models to separately account for large and small vessels [12, 13]. Huyghe and Campen [14] introduced a hierarchically arranged model that segmented arterial, capillary, and venous structures, each governed by distinct macroscopic laws. This model used a hierarchy parameter to seamlessly connect compartments, representing larger vessels in initial compartments and smaller vessels in final compartments. Expanding on this approach, Huyghe and Campen [15] also developed a model for determining anisotropic, heterogeneous permeability and porosity within the Darcy continuum, as well as a model for inter‐compartmental flow that relied on Poiseuille flow assumptions and the hierarchy parameter. Cowin et al. [16] contributed a model for compartmental flow as a function of blood pressure oscillations, while Hyde et al. [17] incorporated pressure and flow coupling based on vessel network data from Poiseuille flow. Nakshatrala et al. [18] developed a mathematical model for interacting compartments based on continuum theory with thermodynamic foundations. Michler et al. [19] introduced a streamlined multicompartment Darcy flow model that considered only transmural permeability as relevant for larger vessels while assuming isotropic permeability in microvessels. More recently, Wittwer et al. [20] developed a Helmholtz potential model for pore pressure in each compartment, defined as a function of local porosity, and provided guidelines for selecting the appropriate Helmholtz potential for various applications.

In parallel, Darcy flow models have gained growing interest for patient‐specific simulations of coronary perfusion. Papanamolis et al. [21] used a Darcy flow model coupled with 1D flow simulations in coronary arteries derived from image‐based and synthetic vasculature for modeling CAD, assuming homogeneous porous properties throughout the myocardium. Similarly, Menon et al. [22] used a Constrained Constructive Optimization method to generate synthetic vasculature and compute homogeneous porous parameters, coupling the Darcy flow model with the flow in arteries by averaging total coronary flow. Di Gregorio et al. [23] advanced this by applying a multicompartment Darcy flow model for simulating CAD and obtained myocardial blood flow under stress conditions. Their approach used Poiseuille flow to estimate porous parameters in different perfusion regimes for both healthy and diseased conditions. While these studies represent significant progress in modeling patient‐specific coronary perfusion, they primarily focus on large vessel pathologies, which can be addressed through patient‐specific vascular network parameterization. However, for pathologies involving the microvasculature, accurate patient‐specific parameterization of the Darcy flow domain itself becomes essential.

To that end, recent studies have aimed to parameterize multicompartment porous models directly from vascular geometry. Cookson et al. [24], for example, employed principal component analysis to compute an anisotropic permeability tensor, achieving pressure distributions consistent with Poiseuille flow outcomes. Hyde et al. [17] assessed model accuracy for various permeability calculations—comparing isotropic, PCA‐derived, and anisotropic models—by obtaining permeability at each continuum point based on representative elementary volume (REV) averaging using the blood vessel geometries. Hyde et al. [10] further advanced this approach, deriving patient‐specific, heterogeneous permeability distributions for left ventricular myocardial perfusion that incorporate discrete arterial data for realistic anatomical modeling. For coronary microvasculature in mice, Smith et al. [25] adapted Poiseuille flow principles to derive permeability along principal directions of capillary alignment, revealing strong anisotropy, with permeability in alignment directions an order of magnitude higher than perpendicular directions. For an artificially generated intramural vessel network, Gregorio et al. [26] applied a hierarchy‐based multicompartment model using Hyde's [17] approach to derive isotropic, homogeneous permeability in the myocardium, obtaining myocardial perfusion data that matched physiological values. Qohar et al. [27] studied a two‐compartment model of myocardial perfusion using modified Poiseuille flow with pressure‐matching at junctions and external pressure effects on blood flow in large arteries. The study emphasized how vascular structure and nonlinear factors, like pressure drops at junctions and vessel elasticity, significantly influence local and global microcirculation. Ebrahem et al. [28] optimized the critical value of the hierarchy parameter for the first compartment, yielding the best results. Montino Palegi et al. [29] applied a multicompartment Darcy flow model for a patient‐specific left ventricle, incorporating clinically obtained epicardial arteries and multiple perfusion regions and found that the heterogeneity of myocardial blood flow observed through clinical methods was well captured by the multi‐region model. A similar multi‐region perfusion model was also used in our recent study [30].

These findings highlight a shift toward multicompartment approaches in modeling myocardial blood perfusion. Multicompartment models were first introduced a few decades ago by Huyghe and Campen [14], but patient‐specific models based on these frameworks have only recently gained attention, driven by advances in capturing the detailed structure of the microvasculature. Many studies still utilize isotropic, homogeneous permeability estimates within porous compartments. Only a few studies employ actual microvascular data for compartmental parameterization [10, 17, 19], and they show that capturing anisotropic, heterogeneous permeability based on realistic vasculature greatly influences perfusion accuracy, producing results closer to fluid dynamics simulations with detailed vasculature. However, these models often assume rigid blood vessels and fail to account for the elastic deformation of blood vessels or their coupling with the deformation of the surrounding tissue—factors that play a significant role in cardiac perfusion [7, 27, 31].

In this study, we introduce a comprehensive, patient‐specific multicompartment Darcy flow model that addresses these gaps. Our formulation generalizes previous steady‐state models to the unsteady regime by incorporating time‐dependent flow dynamics, fluid–structure interaction (FSI) within the blood vessels, and nonlinear hyperelastic deformation of the surrounding myocardium, making the model suitable for simulating unsteady cardiac perfusion. To validate the approach, we also develop a simplified steady‐state formulation that retains elastic vessel deformation. We conduct a systematic investigation into the independent effects of spatial heterogeneity in porous parameters and anisotropy in permeability on myocardial pore pressure distribution across multiple scales—an area that remains underexplored in the current literature. Additionally, we carry out a detailed parametric study to determine the optimal number of vascular compartments needed to accurately capture multiscale coronary flow, balancing model fidelity with computational complexity—an aspect rarely examined in depth. Finally, while few studies assess the ability of such models to capture pathological scenarios like vascular blockages, we demonstrate how our heterogeneous, anisotropic framework can simulate intramyocardial obstructions and their impact on perfusion, highlighting its potential for investigating microvascular disease. This framework aims to provide a detailed, physiologically relevant approach for understanding SVD and enhancing the diagnostic accuracy of microvascular disorders.

## 2 Mathematical Formulation

We analyze the blood flow within the vessels coupled with the nonlinear hyperelastic deformation of the myocardium, modeled as a poroelastic continuum. The poroelastic formulation is designed to allow patient‐specific parameterization. In this section, we first present the mathematical formulation of poroelasticity using a multicompartment Darcy flow model, followed by a method to parameterize the model for a specific case and the boundary conditions used.

### 2.1 Poroelastic Formulation

In this framework, the porosity ϕ represents the ratio of the fluid volume to the total volume of the homogeneous mixture. The homogeneous mixture comprises two continua: a fluid continuum and a solid skeleton continuum. At time t, the volumes occupied by the fluid and skeleton continua are

(1) $$V^{f}\left(t\right)=\int_{\Omega_{t}}\phi \left(x,t\right)dx,\;V^{s}\left(t\right)=\int_{\Omega_{t}}\left(1-\phi \left(x,t\right)\right)dx$$

in which $\Omega_{t}$ is the volume occupied by the homogeneous mixture at time t. The position vector $x=\chi \left(X,t\right)$ represents the current configuration of the skeleton at time t, with X being the reference configuration of the skeleton. For the nonlinear deformation of the homogeneous mixture, the deformation of the medium is considered with respect to the skeleton configuration, and the corresponding deformation gradient is defined by

(2) $$F=\frac{\partial x}{\partial X}$$

The corresponding Lagrangian displacement vector is $u\left(X,t\right)=x-X$.

In this study, the fluid continuum representing blood flow through vessels is divided into multiple compartments. The total porosity ϕ is given in terms of the compartmental porosities, given as

(3) $$\phi \left(x,t\right)=\sum_{i=1}^{n_{c}}\;\phi_{i}\left(x,t\right)$$

where the subscript i represents the compartment number and $n_{c}$ is the total number of compartments. For the coupled nonlinear deformation, the total volumes of the fluid and skeleton compartments at times t and t=0 are expressed in terms of the reference configuration as

(4) $$V^{f}\left(t\right)=\int_{\Omega_{0}}J\sum_{i=1}^{n_{c}}\;\phi_{i}\left(\chi \left(X,t\right),t\right)dX,\;V^{f}\left(0\right)=\int_{\Omega_{0}}\sum_{i=1}^{n_{c}}\;\phi_{i0}\left(X\right)dX$$

(5) $$V^{s}\left(t\right)=\int_{\Omega_{0}}J\left(1-\sum_{i=1}^{n_{c}}\;\phi_{i}\left(\chi \left(X,t\right),t\right)\right)dX,\;V^{s}\left(0\right)=\int_{\Omega_{0}}\left(1-\sum_{i=1}^{n_{c}}\;\phi_{i0}\left(X\right)\right)dX$$

in which $\Omega_{0}$ is the volume occupied by the mixture in the reference configuration and $J\left(X,t\right)=\det\;F$ is the Jacobian determinant of the deformation. Throughout this formulation, the subscript 0 indicates quantities defined in the reference configuration. Accordingly, the porosity of the ith compartment in the reference state is given by $\phi_{i0}\left(X\right)=\phi_{i}\left(\chi \left(X,0\right),0\right)$.

In a poroelastic medium, the skeleton and fluid are typically assumed to be incompressible. However, the homogeneous mixture may exhibit compressibility due to the compliance of blood vessels, which allows for expansion and contraction in response to blood pressure variations. The total mass of the homogeneous mixture at time t is $M\left(t\right)=M^{f}\left(t\right)+M^{s}\left(t\right)$, where the total mass of the fluid compartment $M^{f}$ and the skeleton compartment $M^{s}$ at time t and t=0 are evaluated as

(6) $$M^{f}\left(t\right)=\int_{\Omega_{0}}J\sum_{i=1}^{n_{c}}\;\phi_{i}\left(\chi \left(X,t\right),t\right)\rho^{f}\left(\chi \left(X,t\right),t\right)dX,\;M^{f}\left(0\right)=\int_{\Omega_{0}}\sum_{i=1}^{n_{c}}\;\phi_{i0}\left(X\right)\rho_{0}^{f}\left(X\right)dX$$

(7) $$\begin{array}{c} \;M^{s}\left(t\right)=\int_{\Omega_{0}}J\left(1-\sum_{i=1}^{n_{c}}\phi_{i}\left(\chi \left(X,t\right),t\right)\right)\rho^{s}\left(\chi \left(X,t\right),t\right)dX,\;\\ \;M^{s}\left(0\right)=\int_{\Omega_{0}}\left(1-\sum_{i=1}^{n_{c}}\;\phi_{i0}\left(X\right)\right)\rho_{0}^{s}\left(X\right)dX \end{array}$$

in which $\rho^{s}$ and $\rho^{f}$ are the densities of the fluid and solid compartments, respectively. The terms $\rho_{0}^{f}\left(X\right)=\rho^{f}\left(X,0\right)$ and $\rho_{0}^{s}\left(X\right)=\rho^{s}\left(X,0\right)$ represent their corresponding values in the reference state. The mass conservation of the skeleton gives $\Delta M^{s}\left(t\right)=M^{s}\left(t\right)-M^{s}\left(0\right)=0$. Since the homogeneous mixture is not incompressible, the change in mass of the mixture is nonzero, that is, $\Delta M\left(t\right)=M^{f}\left(t\right)-M^{f}\left(0\right)\neq 0$. The total change in mass of the mixture is expressed in terms of the mass changes in the various fluid compartments $\Delta M_{i}\left(t\right)$, given by, $\Delta M\left(t\right)=\sum_{i=0}^{n_{c}}\;\Delta M_{i}^{f}\left(t\right)$, where $\Delta M_{i}^{f}\left(t\right)=\int_{\Omega_{0}}m_{i}\left(X,t\right)dX$. Here, $m_{i}\left(X,t\right)$ is the Lagrangian added mass for compartment i, which represents the change in mass of the ith fluid compartment per unit reference volume, given by

(8) $$m_{i}\left(X,t\right)=J\phi_{i}\left(\chi \left(X,t\right),t\right)\rho^{f}\left(\chi \left(X,t\right),t\right)-\phi_{i0}\left(X\right)\rho_{0}^{f}\left(X\right)$$

Thus, the total added mass is given as

(9) $$m\left(X,t\right)=\sum_{i=1}^{n_{c}}\;m_{i}\left(X,t\right)$$

Assuming the fluid is incompressible, that is, $\rho^{f}\left(X,t\right)\equiv \rho^{f}\left(X,0\right)=\rho_{0}^{f}\left(X\right)$, the Eulerian form of the mass balance equation for the ith fluid compartment is given as [31]:

(10) $$\frac{\partial \phi_{i}\left(x,t\right)}{\partial t}+\nabla \cdot \left(\phi_{i}\left(x,t\right)v_{i}^{f}\left(x,t\right)\right)=s_{i}\left(x,t\right)$$

in which $v_{i}^{f}\left(x,t\right)$ is the fluid velocity and $s_{i}\left(x,t\right)$ is the volumetric source for the ith compartment. Defining the perfusion velocity for each compartment i as $w_{i}\left(x,t\right)=\phi_{i}\left(x,t\right)\left(v_{i}^{f}\left(x,t\right)-v\left(x,t\right)\right)$, where v is the velocity of the skeleton, the Lagrangian form of the mass balance for each fluid compartment is evaluated as [31]:

(11) $$\frac{{\dot{m}}_{i}^{*}\left(X,t\right)}{J}+\nabla \cdot w_{i}\left(\chi \left(X,t\right),t\right)=S_{i}\left(X,t\right)$$

where $m_{i}^{*}=m_{i}/\rho_{0}^{f}$ is the change in volume of the pore fluid in ith compartment per unit reference volume of the skeleton, ${\dot{m}}_{i}^{*}\left(X,t\right)=\partial m_{i}^{*}\left(X,t\right)/\partial t$ is the material derivative of $m_{i}^{*}$, and $S_{i}\left(X,t\right)$ is the Lagrangian volumetric source. The source term consists of the external source $S_{i}^{\mathrm{ext}}$ and a contribution from the flow between the neighboring compartments, given as

(12) $$S_{i}\left(X,t\right)=S_{i}^{\mathrm{ext}}\left(X,t\right)+\sum_{k=1,k\neq i}^{n_{c}}\beta_{k,i}\left(X,t\right)\left(p_{k}^{\text{pore}}\left(X,t\right)-p_{i}^{\text{pore}}\left(X,t\right)\right)$$

here $\beta_{k,i}\left(X,t\right)$ is the coupling constant for flow from compartment k to i, which is considered spatially heterogeneous and derived from the actual blood vessel flow data to facilitate patient‐specific modeling. Further, for simplicity, we assume that the coupling constant does not change with time, that is, $\beta_{k,i}\left(X,t\right)\equiv \beta_{k,i}\left(X,0\right)=\beta_{k,i,0}\left(X\right)$ representing its value in the reference configuration.

The perfusion velocity in each fluid compartment is modeled using Darcy's law, expressed as

(13) $$w_{i}\left(x,t\right)=-K_{i}\left(\chi^{-1}\left(x,t\right),t\right)\nabla p_{i}^{\text{pore}}\left(\chi^{-1}\left(x,t\right),t\right)$$

where $K_{i}$ is the second order permeability tensor and $p_{i}^{\text{pore}}$ is the pore pressure in the reference configuration. For patient‐specific modeling, the permeability tensor is considered to be anisotropic, spatially varying, and parameterized from actual blood vessel data. It is also assumed to be time‐invariant, such that $K_{i}\left(X,t\right)\equiv K_{i}\left(X,0\right)=K_{i0}\left(X\right)$.

Using Darcy's law (13), (11) is pulled back to the reference configuration [31] to obtain the Lagrangian form,

(14) $${\dot{m}}_{i}^{*}-\nabla_{X}\cdot \left(JF^{-1}K_{i0}F^{-T}\nabla_{X}p_{i}^{\text{pore}}\right)=S_{i}^{\mathrm{ext}}+\sum_{k=1,k\neq i}^{n_{c}}\beta_{k,i,0}\left(p_{k}^{\text{pore}}-p_{i}^{\text{pore}}\right)$$

Note that the coupling between compartments is enforced through the second term on the R.H.S, which represents the flow into compartment i from neighboring compartments k in terms of the pore pressures within the compartments.

For the nonlinear poroelastic deformation of the skeleton, assuming the skeleton is incompressible, that is, $\rho^{s}\left(X,t\right)\equiv \rho^{s}\left(X,0\right)=\rho_{0}^{s}\left(X\right)$, the local mass balance equation is obtained using ((6), (7), (8)) [31] as follows:

(15) $$J-1-\sum_{i=1}^{n_{c}}\;m_{i}^{*}=0$$

Neglecting the body force, the momentum balance for the homogeneous mixture is obtained as:

(16) $$\left(\rho_{0}+m\right)\dot{v}=\nabla_{X}\cdot \left(\mathrm{FS}\right)$$

where $\rho_{0}=\rho_{0}^{f}\phi_{0}+\rho_{0}^{s}\left(1-\phi_{0}\right)$ is the density of the homogeneous poroelastic mixture in the reference configuration, and S is the second Piola‐Kirchhoff stress tensor.

The stress tensor S and the pore pressure $p_{i}^{\text{pore}}$ are obtained from the constitutive law for the poroelastic medium, represented by the total free energy [31], given by

(17) $$\Psi \left(C,p,m_{i}^{*}\right)=W\left(\bar{C}\right)+\sum_{i=1}^{n_{c}}\left(U_{i}^{\mathrm{PV}}\left(m_{i}^{*}\right)+U_{i}^{c}\left(m_{i}^{*}\right)\right)-p\left(J-1-\sum_{i=1}^{n_{c}}m_{i}^{*}\right)$$

in which $C=F^{T}F$ is the right Cauchy‐Green tensor and $\bar{C}=J^{-2/3}C$ represents the distortional Cauchy‐Green strain. The term $W\left(\bar{C}\right)$ denotes the strain energy of the hyperelastic skeleton, $U_{i}^{\mathrm{PV}}$ accounts for the free energy of the pore fluid, capturing changes in pore volume due to blood vessel expansion or contraction in each compartment, $U_{i}^{c}$ is a penalization term to prevent negative values of porosity [31] in each compartment, and p represents the skeleton pressure enforcing the incompressibility of the skeleton. In this study, we use a simple nonlinear constitutive model, the neo‐Hookean model, for the hyperelastic solid skeleton. The free energy of the pore fluid, $U_{i}^{\mathrm{PV}}$, is derived based on a nonlinear tube‐law formulation typically used for coronary vessels. In particular, we employ the expression established in previous studies [7, 32]. The penalization term, $U_{i}^{c}$, follows the formulation presented in our earlier work [31]. Accordingly, the energy terms used in (17) of this study are given by

(18) $$\begin{array}{l} \;W\left(\bar{C}\right)=\frac{G}{2}\left({\bar{I}}_{1}-3\right),\\ U_{i}^{\mathrm{PV}}\left(m_{i}^{*}\right)=\frac{q_{1}}{q_{3}}\exp\left(q_{3}\left(m_{i}^{*}+\phi_{i0}\right)\right)+q_{2}\left(m_{i}^{*}+\phi_{i0}\right)\left[\ln\left(q_{3}\left(m_{i}^{*}+\phi_{i0}\right)\right)-1\right]\\ \;-\left[q_{1}\exp\left(q_{3}\phi_{i0}\right)+q_{2}\ln\left(\phi_{i0}\right)\right]\left(m_{i}^{*}+\phi_{i0}\right),\\ \;U_{i}^{c}\left(m_{i}^{*}\right)=c\;\tan^{-1}\left(\frac{m_{i}^{*}+\phi_{i0}-\phi_{\text{crit}}}{\epsilon }\right) \end{array}$$

in which G is the shear modulus of the skeleton; $q_{1}$, $q_{2}$, and $q_{3}$ are material constants obtained from tube flow for the blood vessels; c is the reference pressure; 0<ϵ≪1; and $\phi_{\text{crit}}$ is the critical value of porosity below which further reduction of porosity is prevented to avoid negative porosity. In this study, we adopt the tube law constants $q_{1}=0.022\mathrm{kPa}$, $q_{2}=1.009\mathrm{kPa}$, and $q_{3}=80$, following the values reported by Lee et al. [7]. We found that setting ϵ=0.001 and $\phi_{\text{crit}}=0.00001$ facilitates the convergence of the nonlinear solver while maintaining the constraint $m_{i}^{*}+\phi_{i0}>\phi_{\text{crti}}$.

With the above choice of free energy, the stress tensor S and the pore pressure $p_{i}^{\text{pore}}$ are obtained as

(19) $$\begin{array}{l} \;S=2\frac{\partial \Psi }{\partial C}={\mathrm{GJ}}^{-2/3}\left(I-\frac{C}{3}\right)-\mathrm{pJ}C^{-1},\\ \;p_{i}^{\text{pore}}=\frac{\partial \Psi }{\partial m_{i}^{*}}=p_{i}^{\mathrm{PV}}+p_{i}^{c}+p,\\ \text{where}\;p_{i}^{\mathrm{PV}}=\frac{dU_{i}^{\mathrm{PV}}}{dm_{i}^{*}}=q_{1}\exp\left(q_{3}\left(m_{i}^{*}+\phi_{i0}\right)\right)+q_{2}\left(m_{i}^{*}+\phi_{i0}\right)\left[\ln q_{3}\left(m_{i}^{*}+\phi_{i0}\right)-1\right]\\ \;-q_{1}\exp\left(q_{3}\phi_{i0}\right)+q_{2}\phi_{i0}\left[\ln\left(q_{3}\phi_{i0}\right)-1\right]\\ \;\text{and}\;p_{i}^{c}=\frac{dU_{i}^{c}}{dm_{i}^{*}}=c\frac{\epsilon^{2}}{\epsilon^{2}+\left(m_{i}^{*}+\phi_{i0}-\phi_{\text{crit}}\right)} \end{array}$$

where $p_{i}^{\mathrm{PV}}$ and $p_{i}^{c}$ represent the pore pressure contributions due to pore fluid volume change and penalization, respectively, and are functions solely of the volume change ratio $m_{i}^{*}$. With this, the Lagrangian form of the mass balance equation (Equation 14) becomes:

(20) $$\begin{array}{c} {\dot{m}}_{i}^{*}-\nabla_{X}\cdot \left[JF^{-1}K_{i0}F^{-T}\left(\nabla_{X}p+\frac{d\left(p_{i}^{\mathrm{PV}}+p_{i}^{c}\right)}{dm_{i}^{*}}\nabla_{X}\left(m_{i}^{*}\right)\right)\right]=\\ S_{i}^{\mathrm{ext}}+\sum_{k=1,k\neq i}^{n_{c}}\beta_{k,i,0}\left(p_{k}^{\text{pore}}-p_{i}^{\text{pore}}\right) \end{array}$$

By defining $K_{i0}^{0}=JF^{-1}K_{i0}F^{-T}$ as the Lagrangian permeability tensor and $K_{i0}^{m}=K_{i0}^{0}\left(d\left(p_{i}^{\mathrm{PV}}+p_{i}^{c}\right)/dm_{i}^{*}\right)$ as the added mass dependent permeability tensor, the above equation is simplified to

(21) $${\dot{m}}_{i}^{*}-\nabla_{X}\cdot \left(K_{i0}^{0}\nabla_{X}p+K_{i0}^{m}\nabla_{X}m_{i}^{*}\right)=S_{i}^{\mathrm{ext}}+\sum_{k=1,k\neq i}^{n_{c}}\beta_{k,i,0}\left(p_{k}^{\text{pore}}-p_{i}^{\text{pore}}\right)$$

The above described formulation is designed for general unsteady poroelastodynamic simulations involving nonlinear deformation of the skeleton, such as the myocardium. To effectively compare the poroelastic results with actual blood flow in small and micro blood vessels, it is necessary to model the one‐dimensional unsteady FSI within these vessels. However, performing such simulations is computationally expensive. Consequently, for verification of our model, we assume steady Poiseuille flow in the blood vessels, as commonly done in prior studies [10, 17].

To evaluate the efficacy of the proposed multicompartment Darcy flow model under various patient‐specific conditions, we compare its steady‐state results with those obtained from Poiseuille flow simulations. This comparison is meaningful because the heterogeneity of blood vessels and the pressure gradients are well represented even in steady‐state conditions. Notably, our steady‐state model accounts for the elastic deformation of the vessels due to fluid‐induced pore pressure and its coupling with the nonlinear solid deformation, distinguishing it from traditional approaches. Our primary objective is to assess how accurately the model captures Darcy flow driven by pressure gradients. This analysis, conducted using a steady‐state problem, provides a robust basis for extending the model to unsteady flow scenarios. For the steady‐state multicompartment Darcy flow model, the fluid mass balance (Equation 20) and the homogeneous mixture momentum balance (Equation 16) are given as

(22) $$\begin{array}{c} \nabla_{X}\cdot \left(K_{i0}^{0}\nabla_{X}p\right)+\nabla_{X}\cdot \left(K_{i0}^{m}\nabla_{X}m_{i}^{*}\right)+S_{i}^{\mathrm{ext}}\\ +\sum_{k=1,k\neq i}^{n_{c}}\beta_{k,i,0}\left(p_{k}^{\text{pore}}-p_{i}^{\text{pore}}\right)=0 \end{array}$$

(23) $$\nabla_{X}\cdot \left(\mathrm{FS}\right)=0$$

### 2.2 Parameterization of the Model

The proposed multicompartment porous flow model is constructed under the assumption that a detailed representation of the coronary microvasculature is available. This vascular data is essential for parameterizing the porosity, permeability tensor, and coupling constants of the porous medium. While such high‐resolution vascular data is challenging to obtain directly from medical imaging in clinical settings, recent advances in vascular generation algorithms provide a viable alternative. In this study, we use the Constrained Constructive Optimization (CCO) algorithm [2] to generate synthetic yet physiologically realistic coronary vessel networks, which serve as the input for porous parameter estimation.

**FIGURE 1:**
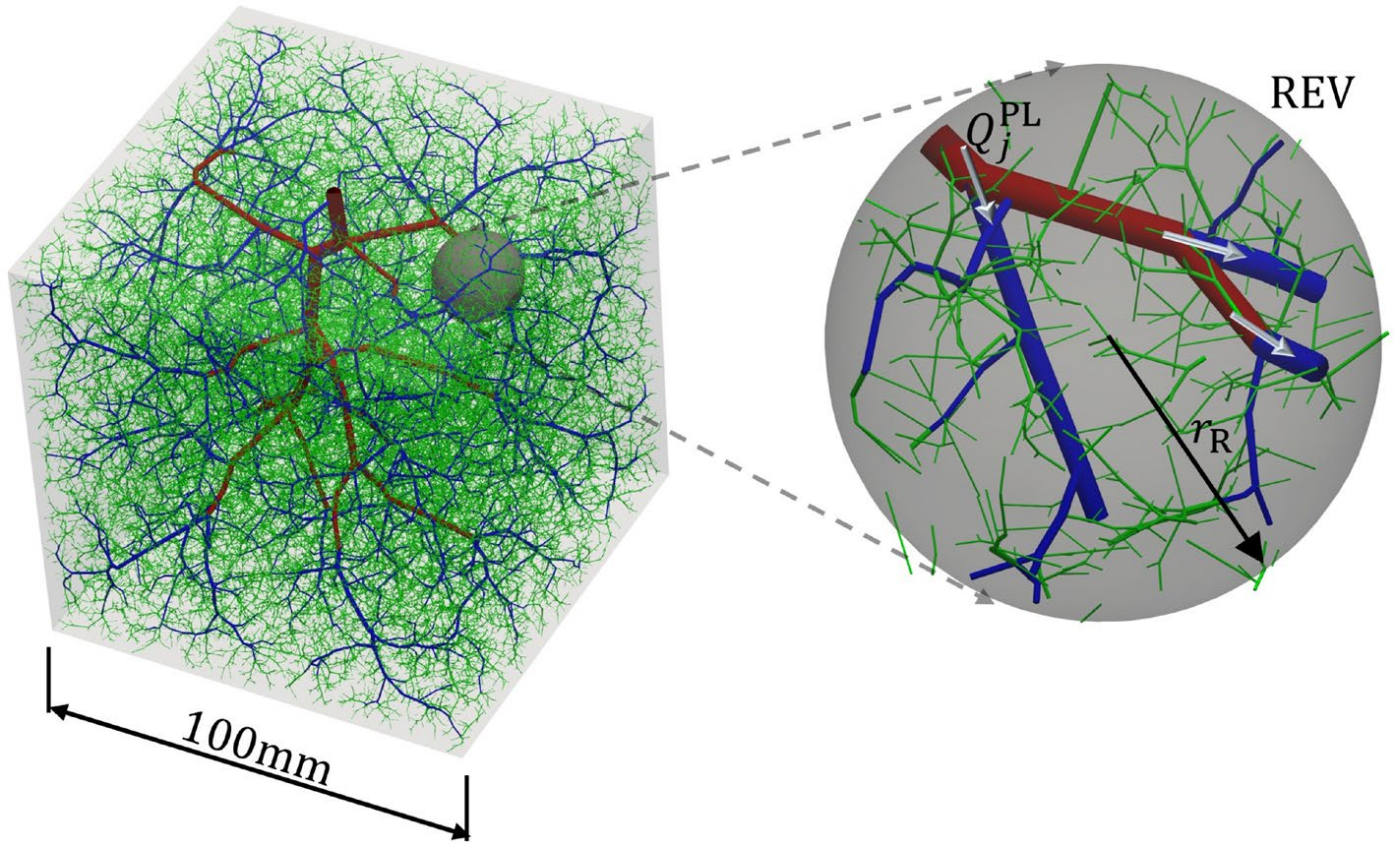
Visualization of blood vessels within a cube generated using the CCO algorithm [3] with 50,000 number of terminal vessels, divided into three compartments (1st‐red, 2nd‐blue, 3rd‐green) based on the hierarchy parameter, and a depiction of flow interactions between compartments within the REV encompassing vessels from the different compartments. The flow between compartments 1 and 2 within the REV, denoted as $Q_{1,2}^{\mathrm{PL}}$, is calculated as the sum of the Poiseuille flow rates $Q_{j}^{\mathrm{PL}}$ from all vessels j in compartment 1 that connect to vessels in compartment 2.

To demonstrate model parameterization, we first consider perfusion within a cubic domain containing an artificially generated vascular network, created using the CCO algorithm. Figure 1 illustrates the computational domain—a cube of size 100 mm—with the embedded vessel network. The network is generated with an inlet vessel radius of 3 mm, and the inlet flow is evenly distributed among 50,000 terminal vessels. The applied boundary conditions are a fixed inlet pressure of 100mmHg and an outlet pressure of 0mmHg. Further details of the CCO method are provided in Appendix A.

The vascular network is divided into multiple compartments, each representing vessels at different hierarchical levels. This approach improves the homogenization of discrete blood vessels into a continuum, reducing heterogeneity. To achieve this, we employ a hierarchy parameter introduced by Huyghe and Campen [15] and subsequently adopted by many researchers [17, 19]. For a vessel j, the hierarchy parameter $\zeta_{j}$ is defined as

(25) $$\zeta_{j}=\frac{l_{D,j}}{l_{T}}$$

where $l_{D,j}$ represents the sum of the lengths of the vessels distal to the vessel j, and $l_{T}$ is the sum of the lengths of all the vessels in the network. Consequently, ζ=1 for the inlet vessel and ζ=0 for the outlet vessel. In the multi‐compartment model, each compartment is defined by a specific range of critical values of the hierarchy parameter. The division into $n_{c}$ number of compartments is carried out with the objective of maximizing homogeneity in vessel radius within each group. To this end, we employ a regression tree algorithm [33], using the hierarchy parameter ζ as the predictor and the vessel radius as the target variable. The regression tree recursively selects critical values of ζ that minimize the within‐group variance of the radius. By constraining the tree to exactly $n_{c}$ terminal nodes, the method partitions the vascular network into $n_{c}$ compartments, each corresponding to a contiguous interval of the hierarchy parameter with relatively uniform vessel size. The critical values defining these compartments are represented by the vector $\left[\zeta_{1}^{c},\zeta_{2}^{c},\ldots ,\zeta_{n_{c}}^{c},\zeta_{n_{c}+1}^{c}\right]$, such that compartment i includes all vessels satisfying $\zeta_{j}\in \left[\zeta_{i}^{c},\zeta_{i+1}^{c}\right]$.

The porous parameters of the model—porosity, permeability, and the coupling constant—are computed at each point in the computational domain by homogenizing discrete blood vessel data in each compartment within a REV [34] surrounding that point. This leads to spatially varying porous parameters within the domain. Figure 1 illustrates a spherical REV centered at a location X in the domain from which these parameters are extracted. The initial porosity at a point X is determined using the REV approach as

(26) $$\phi_{i0}\left(X\right)=\frac{1}{V_{R}}\sum_{j=1}^{n_{v,i}}\;\pi r_{j\left(i\right)}^{2}l_{j\left(i\right)}$$

where r and l represent the radius and length, respectively, and the subscript $j\left(i\right)$ represents the $j^{\mathrm{th}}$ vessel in compartment i within the REV, $n_{v,i}$ represents the total number of blood vessels in compartment i within the REV, and $V_{R}$ is the volume of the REV.

For computing the permeability and the coupling constant, following Hyde et al. [17], we assume that the flow in small vessels is nearly steady and governed by Poiseuille's law. In this study, we examine three models for the permeability, each representing a different level of complexity.

**Model I** assumes that the permeability in each compartment is locally dependent on the vascular structure and is anisotropic. Based on the Poiseuille flow assumption for blood flow in vessels and using the REV homogenization approach [17], the initial permeability tensor $K_{i0}\left(X\right)$ is defined as a heterogeneous and anisotropic quantity

(27) $$K_{i0}\left(X\right)=\sum_{j=1}^{n_{v,i}}\frac{\pi r_{j\left(i\right)}^{4}l_{j\left(i\right)}t_{j\left(i\right)}⊗t_{j\left(i\right)}}{8\mu V_{R}}$$

in which $t_{j\left(i\right)}$ is the unit vector along the vessel j in $i^{\mathrm{th}}$ compartment, and μ is the viscosity of the blood.

**Model II** simplifies the assumption by considering the permeability tensor to be isotropic, while still spatially heterogeneous. The initial permeability tensor within the REV is then expressed as

(28) $$K_{i0}\left(X\right)=\sum_{j=1}^{n_{v,i}}\frac{\pi r_{j\left(i\right)}^{4}l_{j\left(i\right)}}{8\mu V_{R}}I$$

where I is the identity tensor.

**Model III** further simplifies the formulation by assuming that the porous parameters are homogeneous across each compartment. In this case, the average permeability tensor over the entire domain is given by

(29) $$K_{i0}=\sum_{j=1}^{N_{v,i}}\frac{\pi r_{j\left(i\right)}^{4}l_{j\left(i\right)}}{8\mu V_{T}}I$$

where $N_{v,i}$ is the total number of vessel in ith compartment segments across the entire domain and $V_{T}$ is the total volume of the computational domain.

The flow between compartments $Q_{k,i}\left(X\right)$ at any location X within the REV is given as the sum of flow entering vessels in compartment i at junctions connecting to vessels in compartment k. Figure 1 illustrates an example of such an interaction between compartments 1 and 2. This approach applies when discrete flow rates within the vessels are known. However, in the continuum formulation based on Darcy's law, the inter‐compartmental flow is modeled as being proportional to the pressure difference between the compartments within the REV, as described in (12). Accordingly, the flow rate $Q_{k,i}\left(X\right)$ and the coupling constant $\beta_{k,i,0}\left(X\right)$ are related by

(30) $$\beta_{k,i,0}\left(X\right)=\frac{Q_{k,i}\left(X\right)}{V_{R}\left(p_{k}^{\text{pore}}\left(X\right)-p_{i}^{\text{pore}}\left(X\right)\right)}$$

where $p_{k}^{\text{pore}}$ and $p_{i}^{\text{pore}}$ are the fluid pressures within the REV in compartments k and i, respectively. In practice, the coupling constant is often assumed to be a uniform value across the domain [7]. In this study, following the approach of Hyde et al. [17], we estimate the coupling constant directly from vascular data to enable patient‐specific parameterization. For this purpose, we assume steady‐state flow governed by Poiseuille's law. Despite its simplicity, this assumption provides a more accurate and spatially varying parameterization than using a constant value. Consequently, for Models I and II, the coupling constant is approximated as

(31) $$\beta_{k,i,0}\left(X\right)=\frac{Q_{k,i}^{\mathrm{PL}}\left(X\right)}{V_{R}\left|{\bar{p}}_{k}^{\mathrm{PL}}\left(X\right)-{\bar{p}}_{i}^{\mathrm{PL}}\left(X\right)\right|}$$

in which $Q_{k,i}^{\mathrm{PL}}$ is the Poiseuille flow rate from compartment k to i within the REV, and ${\bar{p}}_{k}^{\mathrm{PL}}$ and ${\bar{p}}_{i}^{\mathrm{PL}}$ are the corresponding average Poiseuille pressures in compartments k and i, respectively. For any compartment i, the average Poiseuille pressure within an REV is computed as

(32) $${\bar{p}}_{i}^{\mathrm{PL}}\left(X\right)=\frac{1}{V_{R}}\sum_{j=1}^{n_{v,i}}\;\pi r_{j\left(i\right)}^{2}l_{j\left(i\right)}p_{j\left(i\right)}^{\mathrm{PL}}$$

where $p_{j\left(i\right)}^{\mathrm{PL}}$ is the average Poiseuille pressure in vessel j from compartment i within the REV.

For Model III, the coupling constant is assumed to be uniform across the domain and is based on the total flow rate from vessels in compartment k to i, $Q_{k,i,\mathrm{tot}}^{\mathrm{PL}}$, along with the spatially averaged pressures, ${\bar{p}}_{k,\mathrm{avg}}^{\mathrm{PL}}$ and ${\bar{p}}_{i,\mathrm{avg}}^{\mathrm{PL}}$ over the entire domain, given as

(33) $$\beta_{k,i,0}=\frac{Q_{k,i,\mathrm{tot}}^{\mathrm{PL}}}{V_{R}\left|{\bar{p}}_{k,\mathrm{avg}}^{\mathrm{PL}}-{\bar{p}}_{i,\mathrm{avg}}^{\mathrm{PL}}\right|}$$

The average Poiseuille pressure in compartment i is

(34) $${\bar{p}}_{i,\mathrm{avg}}^{\mathrm{PL}}=\frac{1}{V_{R}}\sum_{j=1}^{N_{v,i}}\;\pi r_{j\left(i\right)}^{2}l_{j\left(i\right)}p_{j\left(i\right)}^{\mathrm{PL}}$$

To determine the discrete values of Poiseuille flow rates and pressures in each vessel of the complete vascular network, we solve the steady‐state flow equations based on the Poiseuille flow assumption combined with flow balance constraints. This approach is computationally efficient and significantly less demanding than the full unsteady FSI simulations that require much finer spatial and temporal discretizations. The detailed procedure for computing Poiseuille flow and pressure throughout the network is provided in Appendix B.

### 2.3 Boundary Conditions

For the multicompartment Darcy flow model, following previous perfusion studies [19, 31], the boundary surface of the domain is assumed to be impermeable. In addition, we neglect any external forces that act on the surface of the body. Thus, the boundary conditions are

(35) $$\begin{array}{r} W_{i}\cdot dA=-\left(K_{i0}^{0}\nabla_{X}p+K_{i0}^{m}\nabla_{X}m_{i}^{*}\right)\cdot dA=0,\;X\in \Gamma_{0},\\ t_{0}=0,\;X\in \Gamma_{0} \end{array}$$

where $W_{i}$ is the Lagrangian perfusion velocity, dA is the elemental area vector at the boundary $\Gamma_{0}$ of the computational domain $\Omega_{0}$ in the reference configuration, and $t_{0}$ is the external traction vector acting on the surface $\Gamma_{0}$.

For the Poiseuille flow solution in the vascular network, we impose prescribed pressure boundary conditions at the inlet and outlet vessels, denoted by $p^{\text{in}}$ and $p^{\text{out}}$, respectively (Appendix B). To couple the discrete blood flow in large vessels with the continuum Darcy model, the pressure in the first compartment, $p_{1}^{\text{pore}}$, is derived from the Poiseuille pressure field via volume averaging over the REV

(36) $$p_{1}^{\text{pore}}\left(X\right)={\bar{p}}_{1}^{\mathrm{PL}}\left(X\right)$$

For the last compartment, the boundary condition is imposed using the external source term $S_{n_{c}}^{\mathrm{ext}}$ derived from the Poiseuille flow within the REV, expressed as

(37) $$S_{n_{c}}^{\mathrm{ext}}\left(X\right)=\frac{1}{V_{R}}\sum_{t=1}^{n_{t}}\;Q_{t}^{\mathrm{PL}}$$

in which $Q_{t}^{\mathrm{PL}}$ denotes the Poiseuille flow rate in the tth terminal vessel and $n_{t}$ is the total number of terminal vessels within the REV.

## 3 Numerical Formulation

The governing equations for the multicompartment Darcy flow coupled with nonlinear deformation are solved numerically using the Galerkin Finite Element method. The corresponding weak formulation of the Lagrangian mass balance equation of the pore fluid and the momentum balance for the homogeneous mixture is given as

(38) $$\begin{array}{r} \int_{\Omega_{0}}\left(K_{i0}^{0}\nabla_{X}p+K_{i0}^{m}\nabla_{X}m_{i}^{*}\right)\cdot \nabla_{X}{\tilde{m}}_{i}dX-\int_{\Omega_{0}}S_{i}{\tilde{m}}_{i}dX+\int_{\Gamma_{0}}W_{i}\cdot {\tilde{m}}_{i}dA=0,\\ \int_{\Omega_{0}}\left(\mathrm{FS}\right)\cdot \nabla_{X}\tilde{u}dX-\int_{\Gamma_{0}}t_{0}\cdot \tilde{u}dA=0 \end{array}$$

in which ${\tilde{m}}_{i}$ and $\tilde{u}$ are the test functions corresponding to the variables $m_{i}^{*}$ and u, respectively. Using the boundary conditions (35), the complete weak formulation of the problem is given as

(39) $$\begin{array}{r} \int_{\Omega_{0}}\left(K_{i0}^{0}\nabla_{X}p+K_{i0}^{m}\nabla_{X}m_{i}^{*}\right)\cdot \nabla_{X}{\tilde{m}}_{i}dX-\int_{\Omega_{0}}S_{i}{\tilde{m}}_{i}dX=0,\\ \int_{\Omega_{0}}\left(\mathrm{FS}\right)\cdot \nabla_{X}\tilde{u}dX=0,\\ \int_{\Omega_{0}}\left(J-1-\sum_{i=1}^{n_{c}}\;m_{i}^{*}\right)\tilde{p}dX=0 \end{array}$$

where $\tilde{p}$ is the test function corresponding to p.

To achieve computational efficiency, we employ second‐order accurate piecewise linear (P1) finite elements for the formulation [31]. However, due to the incompressibility condition of the skeleton, using the same interpolation order for displacement and pressure violates the Ladyzhenskaya‐Babuška‐Brezzi (LBB) criterion [35], causing numerical instability. Since higher‐order finite elements are computationally expensive, we retain linear elements and address the instability with an efficient stabilized finite element method [31, 36, 37].

In this stabilization approach, the residual of the mass balance equation is augmented with a small perturbation term derived from a fine‐scale component of the displacement vector, $u^{\prime }$. This modification stabilizes the equations, as expressed by

(40) $$\begin{array}{r} \int_{\Omega_{0}}\left(J-1-\sum_{i=1}^{n_{c}}\;m_{i}^{*}\right)\tilde{p}\;dX+\sum_{K\in T}\int_{K}\left(u^{\prime }\cdot \left(JF^{-T}\nabla_{X}^{*}\tilde{p}\right)\right)dX=0,\\ \text{where},u^{\prime }=-\frac{\alpha h^{2}}{2G}F^{-T}\nabla_{X}p \end{array}$$

in which K is the element of triangulation T, such that $\bar{\Omega }=\cup_{K\in T}K$ [37]. For the three‐dimensional model considered in this study, K represents tetrahedral elements. Furthermore, h denotes the characteristic length of the element, and α is a non‐negative, dimensionless stabilization parameter dependent on the element type [36]. Numerical experiments revealed that the method achieves stabilization without introducing excessive dissipative effects that could significantly influence the results when α is set within the range 0.1≤α≤0.3. The stabilized weak form of the governing equations leads to a set of nonlinear equations, which are solved using Newton's method. In practice, the nonlinear solver typically converges within five Newton iterations.

## 4 Results

**FIGURE 2:**
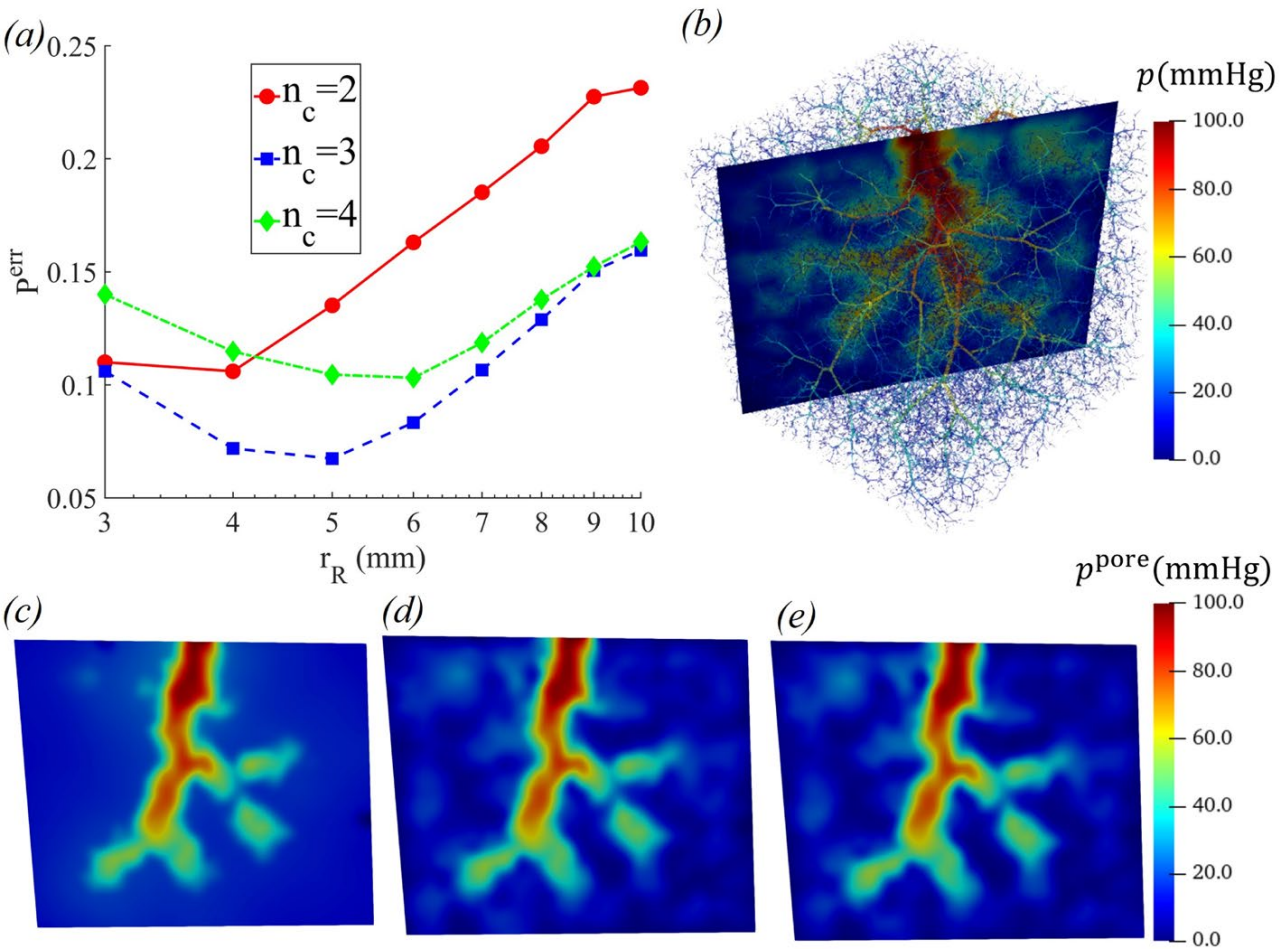
(a) Variation of pore pressure error, $P^{\mathrm{err}}$, with REV radius for $n_{c}=2$, $n_{c}=3$, and $n_{c}=4$; (b) Poiseuille flow pressure distribution in a two‐dimensional plane within the cube; and pore pressure distributions in the two‐dimensional plane for (c) $n_{c}=2$, (d) $n_{c}=3$, and (e) $n_{c}=4$.

To evaluate the effectiveness of the proposed multicompartment Darcy flow model with patient‐specific parameterization, we compare results obtained using Models I to III. Numerical simulations are conducted for two representative test cases: (1) perfusion within a cubic domain containing an artificially generated vascular tree constructed using the CCO algorithm (Figure 1), and (2) perfusion within the left ventricle of a porcine heart, where the vascular network is reconstructed using a combination of imaging data and the CCO algorithm.

As the simulations are carried out under steady‐state conditions, the pressure distribution in the discrete vessel network is expected to follow Poiseuille flow. Therefore, to evaluate the fidelity of the continuum model in capturing spatial pressure variations, we compare the continuum pressure field with the discrete Poiseuille pressures. Specifically, the Darcy pressure is evaluated at the center point $X_{j}$ of each vessel segment using interpolation from the finite element solution. The agreement between the two models is quantified via the normalized root mean square error in pore pressure

(41) $$P^{\mathrm{err}}=\sqrt{\frac{1}{N_{v}}\sum_{j=1}^{N_{v}}{\left(\frac{p^{\text{pore}}\left(X_{j}\right)-p_{j}^{\mathrm{PL}}}{p^{\text{in}}}\right)}^{2}}$$

where $N_{v}$ is the total number of vessel segments, $p_{j}^{\mathrm{PL}}$ is the mean Poiseuille pressure along vessel j, and $p^{\text{pore}}\left(X_{j}\right)$ denotes the continuum Darcy pressure evaluated at the midpoint of vessel j, given by

(42) $$p^{\text{pore}}\left(X_{j}\right)=p\left(X_{j}\right)+p_{i}^{\mathrm{PV}}\left(X_{j}\right)+p_{i}^{c}\left(X_{j}\right)$$

with i representing the compartment to which the jth vessel belongs. The inlet pressure $p^{\text{in}}$ is used for normalization.

In the following sections, we present results for both the cubic and ventricular domains in separate subsections. For both cases, we first solve the Poiseuille flow in the discrete vessel network using boundary conditions $p^{\text{in}}=100\;\text{mmHg}$ and $p^{\text{out}}=0\;\text{mmHg}$. The porous medium parameters are then derived via the parameterization procedure described in Section 2.2. Using these parameters, we solve the multicompartment Darcy problem to obtain the continuum pressure distribution. Finally, the Darcy pressure is interpolated at vessel midpoints and compared to the Poiseuille solution using the error metric defined in Equation (41).

### 4.1 Perfusion Inside a Cube

The computational domain for the perfusion inside the cube is shown in Figure 1. We first conduct numerical simulations for a healthy case, where the generated blood vessel network corresponds to a healthy condition. We examine the effects of using different numbers of compartments with varying REV radius, ranging from $r_{R}=3\;\mathrm{mm}$ to $r_{R}=10\;\mathrm{mm}$. We perform numerical simulations for three different values of the number of compartments: $n_{c}=2$, $n_{c}=3$, and $n_{c}=4$. For the simulations, the critical values of $\zeta^{c}$ are obtained from the regression tree algorithm as follows: $\zeta^{c}=\left[1,0.03,0\right]$ for $n_{c}=2$, $\zeta^{c}=\left[1,0.03,0.0003,0\right]$ for $n_{c}=3$, and $\zeta^{c}=\left[1,0.03,0.0008,0.0002,0\right]$ for $n_{c}=4$. Next, we evaluate the accuracy of the porous parameterization by comparing multicompartment Darcy flow results from three approaches (Model I–III). Finally, we demonstrate the significance of the multicompartment Darcy flow model for a special case where one of the large vessels is blocked.

Figure 2a shows the variation of error in pore pressure with REV radius, $r_{R}$, for various numbers of compartments. It is clear from the figure that, for all compartment sizes, the error decreases initially with increasing $r_{R}$, reaches a minimum at an optimal value, and then increases. This is because smaller $r_{R}$ is inadequate for representing the system as a continuum, while a larger $r_{R}$ results in excessive averaging, which overlooks the heterogeneity in the pressure distribution. The optimal $r_{R}$ increases with the number of compartments, as a larger $r_{R}$ is needed to accommodate more discrete vessels within each compartment for higher $n_{c}$. Additionally, the minimum error is smaller for a moderate value of $n_{c}=3$ compared to the smaller value of $n_{c}=2$, since the heterogeneity of the porous parameters in both large and small blood vessels is more accurately accounted for and scaled for $n_{c}=3$. However, as $n_{c}$ increases to 4, the error increases again. This is due to the need for more coupling constants ($\beta_{k,i,0}$), which are derived through interpolation from Poiseuille flow results. As the number of compartments increases, the interpolation error also increases, leading to a larger error in pore pressure.

**FIGURE 3:**
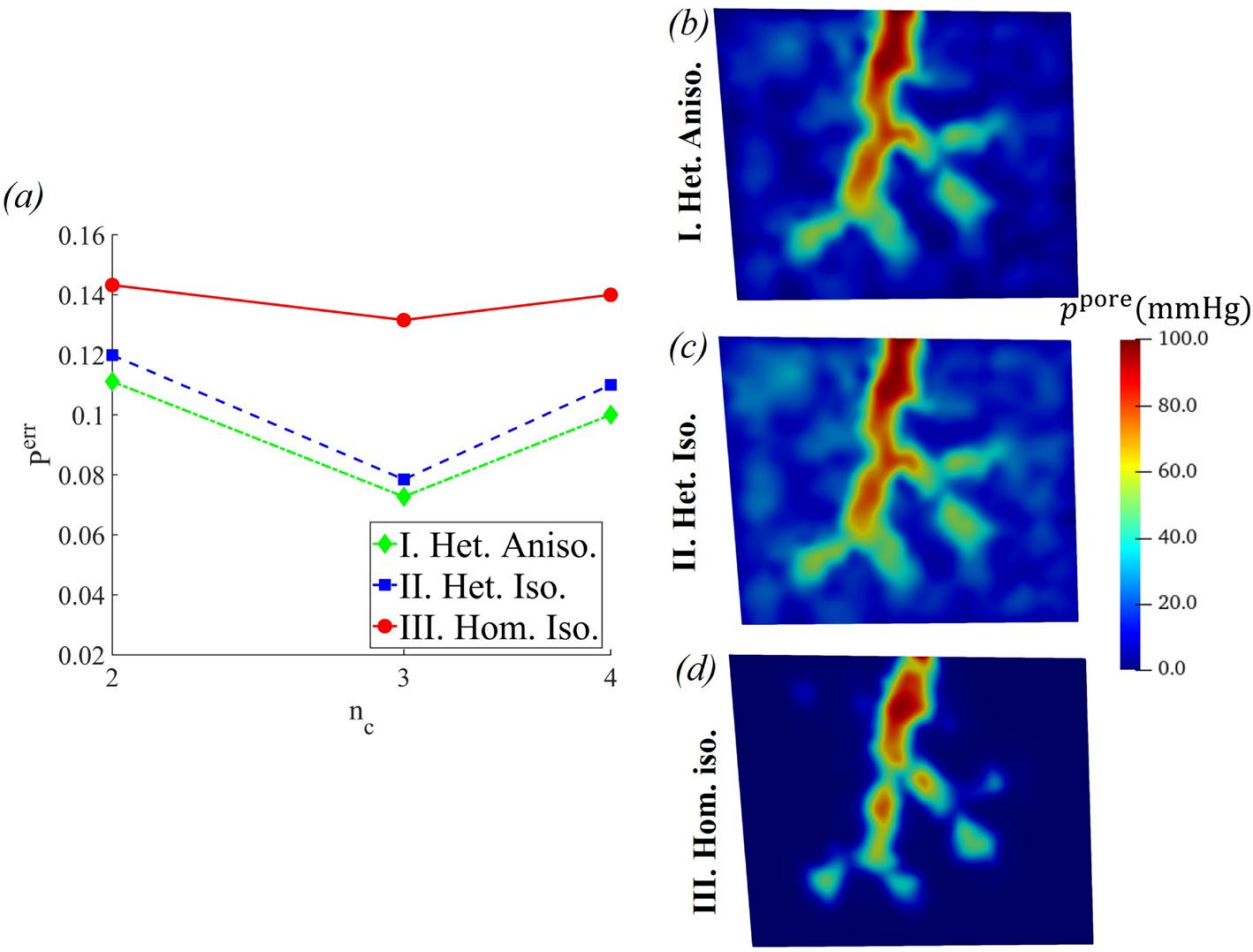
(a) Variation of pore pressure error $P^{\mathrm{err}}$ with the number of compartments $n_{c}$ for the heterogeneous anisotropic (Model I), heterogeneous isotropic (Model II), and homogeneous (Model III) models along with the (b–d) pore pressure distribution in a two‐dimensional plane in the cube obtained with $n_{c}=3$ for the different models.

Figure 3a shows the variation in the error of pore pressure with $n_{c}$, obtained from the various models (Model I—III). The smaller errors for the heterogeneous models (Models I and II) indicate that the heterogeneity of the porous parameters has a significant impact on pore pressure prediction. Figure 3b–d reveals that the pore pressure in the small vessels is more accurately captured in $n_{c}=3$. Furthermore, Table 1 demonstrates that the porous parameters exhibit considerable heterogeneity across all the different compartments.

**TABLE 1:** Average values of the porous parameters across the entire computational domain in different compartments for various numbers of compartments.

Parameter	$n_{c}=2$		$n_{c}=3$			$n_{c}=4$			
	1st comp.	2nd comp.	1st comp.	2nd comp.	3rd comp.	1st comp.	2nd comp.	3rd comp.	4th comp.
Porosity	0.022	0.0026	0.022	0.0017	0.0015	0.022	0.0022	0.0005	0.0014
	±0.025	±0.002	±0.026	±0.0023	±0.0004	±0.026	±0.0026	±0.0004	±0.0004
Isotropic permeability	1.0e‐3	3.9e‐6	1.0e‐3	5.2e‐6	2.9e‐7	1.0e‐3	9.0e‐6	3.4e‐7	2.3e‐7
	±2.2e‐3	±1.1e‐5	±2.2e‐3	±1.3e‐5	±1.5e‐7	±2.2e‐3	±1.7e‐5	±3.2e‐7	±1.1e‐7
Coupling constant	4.3e‐5	1.5e‐6	9.0e‐5	1.5e‐6	1.7e‐6	1.1e‐4	5.8e‐6	1.3e‐6	1.8e‐6
	±6.0e‐5	±9.8e‐7	±1.2e‐4	±1.0e‐6	±9.1e‐7	±1.6e‐4	±1.9e‐5	±9.6e‐7	±9.4e‐7

**FIGURE 4:**
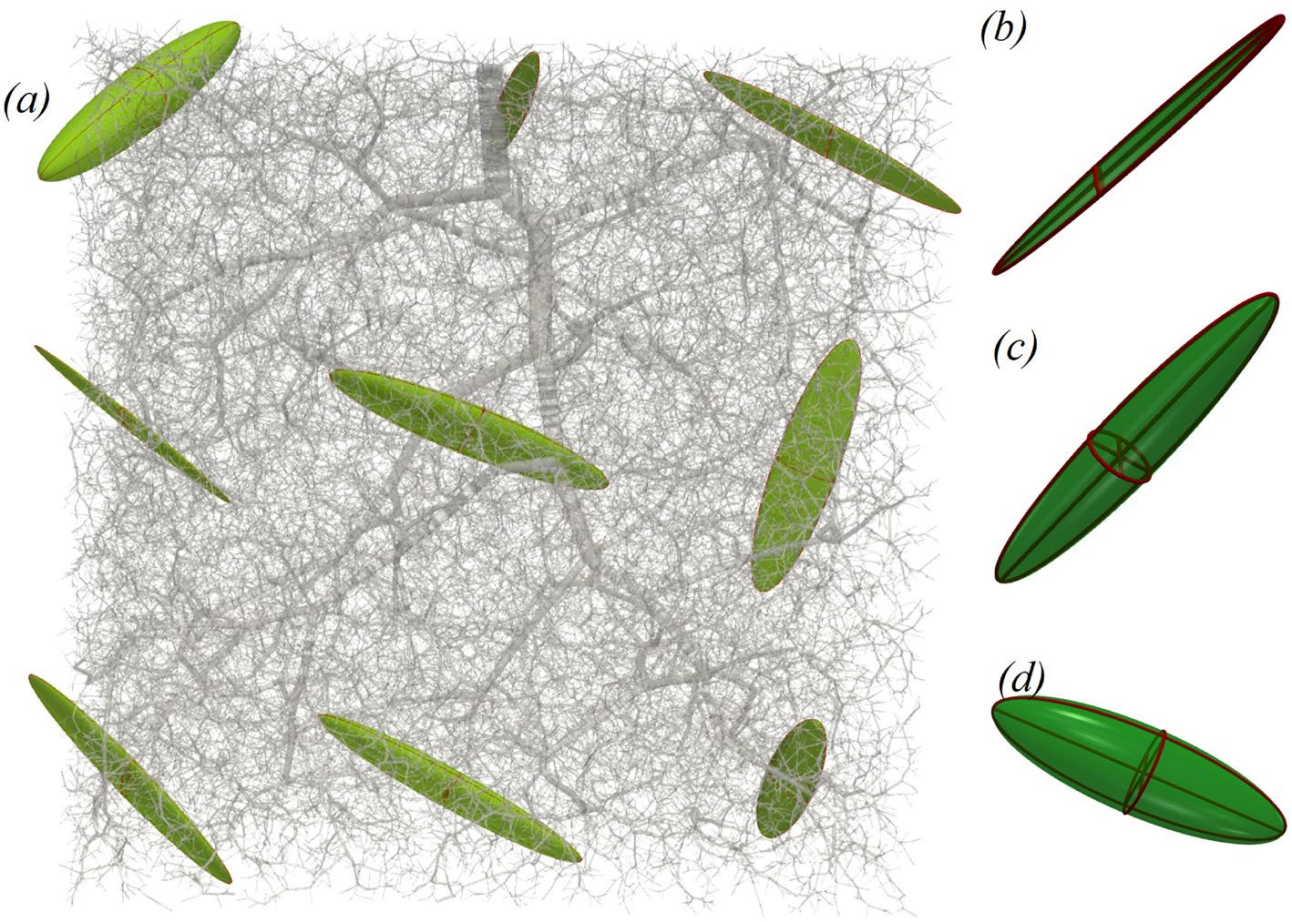
Ellipsoidal representation of (a) the principal components of the permeability tensor at different locations within the second compartment and the space‐averaged permeability tensor across the whole cube in (b) the first compartment, (c) the second compartment, and (d) the third compartment for the $n_{c}=3$ model.

Figure 3 also shows that the model with anisotropic permeability (Model I) results in a slightly smaller error than the isotropic model (Model II). However, relative to the impact of porous parameter heterogeneity, the improvement due to permeability anisotropy is only marginal. Figure 4 further illustrates that the permeability exhibits notable anisotropy, with the highest degree of anisotropy in the first compartment, gradually decreasing in the final compartment. The capillary vessels exhibit almost an isotropic behavior [19]. Table 2 shows the heterogeneity and anisotropy of average permeabilities in the principal directions for different compartments. Note from Tables 1 and 2 that the standard deviation of the poroelastic parameters in the first compartment exceeds its mean value, indicating a high degree of variability. The parameters are nearly zero in most elements, and homogenization is not recommended. Thus, our simulations treat the pressure in the first compartment as boundary conditions derived from Poiseuille flow (36).

**TABLE 2:** Average values of the principal components of the permeability tensor ($k_{1}$, $k_{2}$, and $k_{3}$) across the whole domain in different compartments for various numbers of compartments.

Permeability	$n_{c}=2$		$n_{c}=3$			$n_{c}=4$			
	1st comp.	2nd comp.	1st comp.	2nd comp.	3rd comp.	1st comp.	2nd comp.	3rd comp.	4th comp.
$k_{1}$	5.7e‐6	8.6e‐8	5.7e‐6	8.0e‐8	2.1e‐8	5.7e‐6	1.1e‐7	8.2e‐9	1.8e‐8
	±2.5e‐5	±2.1e‐7	±2.5e‐5	±2.5e‐7	±1.1e‐8	±2.5e‐5	±3.1e‐7	±1.4e‐8	±9.0e‐9
$k_{2}$	6.1e‐5	3.4e‐7	6.1e‐5	4.1e‐7	4.0e‐8	6.1e‐5	6.7e‐7	3.4e‐8	3.2e‐8
	±2.1e‐4	±1.1e‐6	±2.1e‐4	±1.4e‐6	±2.3e‐8	±2.1e‐4	±1.8e‐6	±4.4e‐8	±1.7e‐8
$k_{3}$	9.7e‐4	3.5e‐6	9.7e‐4	4.8e‐6	2.3e‐7	9.7e‐4	8.2e‐6	2.9e‐7	1.8e‐7
	±2.0e‐3	±1.0e‐5	±2.0e‐3	±1.2e‐5	±1.2e‐7	±2.0e‐3	±1.6e‐5	±2.8e‐7	±9.3e‐8

**FIGURE 5:**
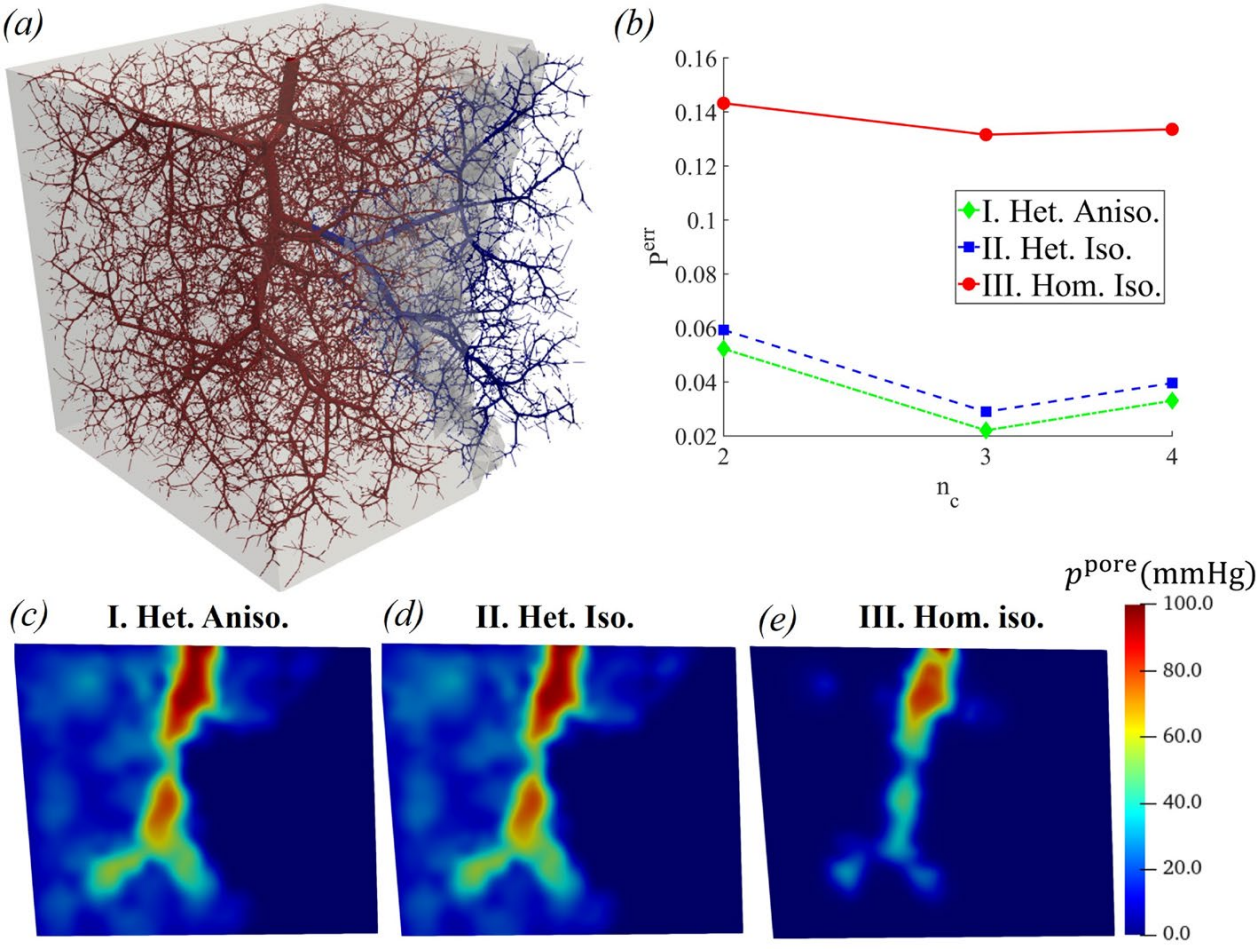
(a) Blood vessels in the cube with one of the vessel branches blocked (blue), and the corresponding (b) variation of pore pressure error $P^{\mathrm{err}}$ with the number of compartments $n_{c}$ for the heterogeneous anisotropic (Model I), heterogeneous isotropic (Model II), and homogeneous (Model III) models, along with the (c–e) pore pressure distribution in a two‐dimensional plane in the cube obtained with $n_{c}=3$ for the different models.

Figure 5 shows the results for perfusion with one of the large vessels blocked, representing a diseased case. The homogeneous model (Model III), which assumes average permeability throughout the domain, fails to account for the impermeable regions (Figure 5e), whereas the heterogeneous models (Models I and II) accurately capture these regions (Figure 5c,d). As a result, the homogeneous model (Model III) produces a larger error in pore pressure, leading to a greater difference in the error between the homogeneous and heterogeneous models compared to the healthy case.

### 4.2 Perfusion Inside a Left Ventricle

**FIGURE 6:**
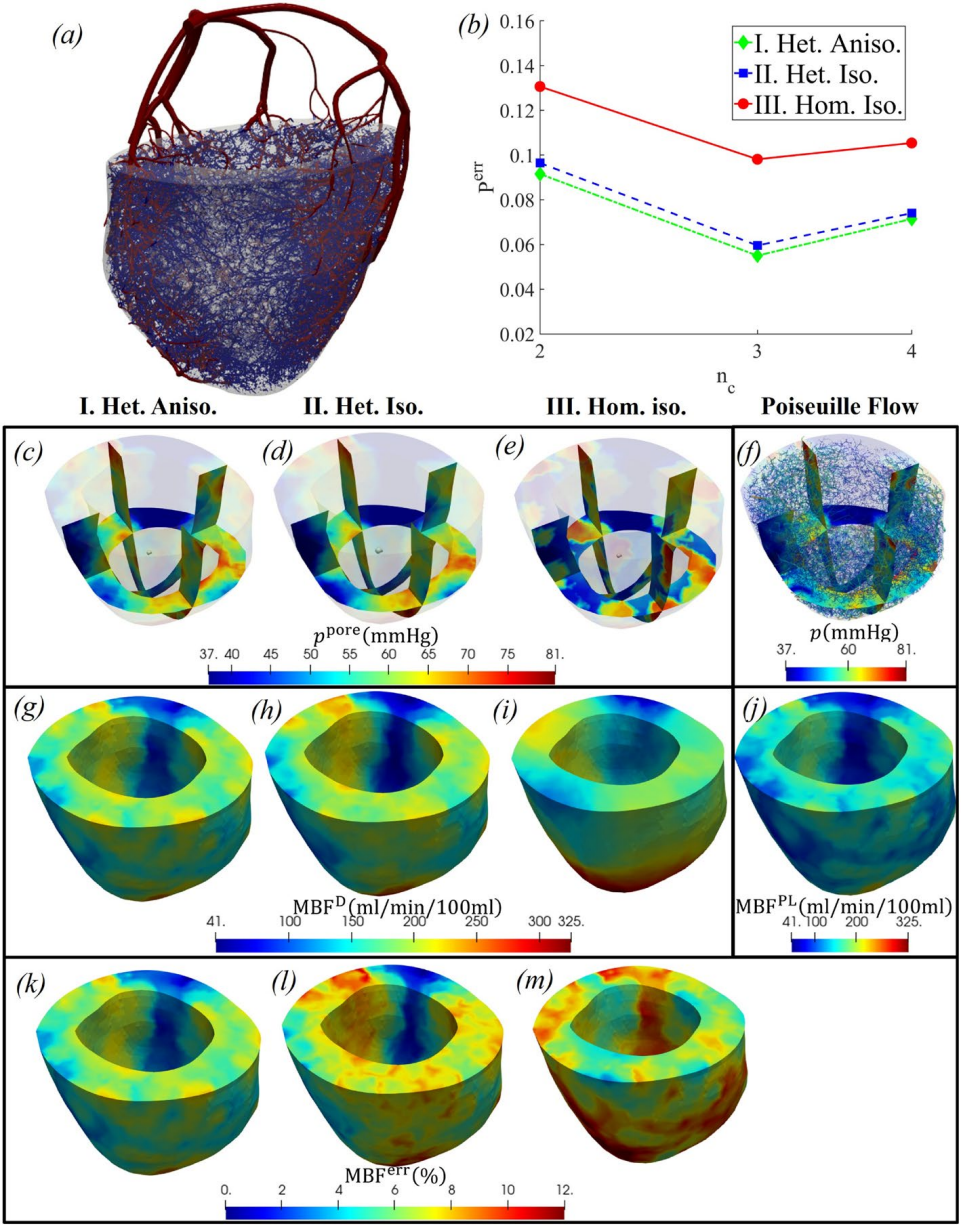
(a) Left ventricular myocardium of a porcine heart with coronary vessels obtained from imaging (red) and generated using the CCO algorithm (blue); (b) variation of pore pressure error $P^{\mathrm{err}}$ with the number of compartments $n_{c}$; (c–e) fields of pore pressure distributions from Models I–III in selected myocardial cross‐sections; (f) Poiseuille pressure in the vascular network and its interpolation within myocardial cross‐sections; (g–i) fields of Darcy‐based myocardial blood flow ${\mathrm{MBF}}^{D}$ for Models I–III; (j) field of Poiseuille‐based myocardial blood flow ${\mathrm{MBF}}^{\mathrm{PL}}$; and (k–m) relative error fields ${\mathrm{MBF}}^{\mathrm{err}}$ for Models I–III. Results in panels (c)–(m) correspond to $n_{c}=3$.

Figure 6a presents the computational domain used for simulating perfusion within the left ventricle (LV) of a porcine heart, based on a previously developed image‐derived geometry [7]. The coronary vasculature, including vessels of diameter down to 0.1 mm, was reconstructed from imaging data and utilized in earlier studies [38]. However, for the present study, a complete vascular network is required. To this end, the remaining smaller vessels were generated using the CCO algorithm.

The CCO algorithm, implemented in a manner similar to that described in Appendix A, was adapted to generate the microvasculature data. Specifically, the larger vessel tree served as the initial condition for the CCO generation, following the methodology outlined in Jaquet et al. [39] for integrating anatomical vascular structures with synthetic tree growth. Boundary conditions for the CCO algorithm were set with an inlet radius of 1.7mm. As with the cubic perfusion case, we conducted simulations over a range of REV radii $r_{R}$ and compartment numbers $n_{c}$. The critical values of $\zeta^{c}$ used for compartmental division were $\zeta^{c}=\left[1,0.03,0\right]$ for $n_{c}=2$, $\zeta^{c}=\left[1,0.03,0.0003,0\right]$ for $n_{c}=3$, and $\zeta^{c}=\left[1,0.03,0.0008,0.0002,0\right]$ for $n_{c}=4$. The optimal REV size was determined to be $r_{R}=2\;\mathrm{mm}$ based on the minimum pressure error $P^{\mathrm{err}}$. All subsequent LV simulations were therefore performed using $r_{R}=2\;\mathrm{mm}$.

Figure 6b presents the error in pore pressure predicted by the three models for varying numbers of compartments. As observed in the cubic case, the heterogeneous model (Model II) yields more accurate results than the homogeneous model (Model III), while the anisotropic model (Model I) offers a slight additional improvement. The lowest error was observed for $n_{c}=3$ compartments. In addition to pore pressure, we also evaluated a quantity analogous to the myocardial blood flow (MBF) [23]. The MBF derived from the Darcy‐based continuum model, ${\mathrm{MBF}}^{D}$, is computed as the volumetric flow into the final compartment

(43) $${\mathrm{MBF}}^{D}\left(X\right)=\beta_{n_{c}-1,n_{c},0}\left(X\right)\left[p_{n_{c}-1}^{\text{pore}}\left(X\right)-p_{n_{c}}^{\text{pore}}\left(X\right)\right]$$

This continuum‐based estimate was compared with the MBF calculated from the discrete Poiseuille flow model, ${\mathrm{MBF}}^{\mathrm{PL}}$, defined as

(44) $${\mathrm{MBF}}^{\mathrm{PL}}\left(X\right)=\frac{1}{V_{R}}Q_{n_{c}-1,n_{c}}^{\mathrm{PL}}$$

where $Q_{n_{c}-1,n_{c}}^{\mathrm{PL}}$ is the Poiseuille flow rate from compartment $n_{c}-1$ to $n_{c}$ within the REV. We quantified the relative error between the two MBF estimates as ${\mathrm{MBF}}^{\mathrm{err}}$, and the corresponding results are shown in the Figure 6. The results show that the heterogeneous (Model II) and anisotropic (Model I) models yield more accurate MBF predictions than the homogeneous model (Model III).

### 4.3 Discussion

The parameters obtained through the proposed approach are highly dependent on the geometry of the perfusion domain and the underlying microvascular architecture. As illustrated in Table 3, the coupling constant $\beta_{1,2,0}$ obtained for a three‐compartment model shows notable variation compared to values reported in the literature, reflecting the case‐specific nature of these parameters. Our results further demonstrate that spatial heterogeneity in these parameters within the LV has a significant impact on myocardial perfusion, highlighting the limitations of using spatially uniform values when detailed vascular data are unavailable. The proposed framework, which yields a heterogeneous distribution of porous parameters based on actual vascular networks, is therefore most effective when the full microvascular structure is available.

**TABLE 3:** Comparison of the coupling parameter $\beta_{1,2,0}$ from the proposed model with values reported in the literature.

	LV type	$\beta_{1,2,0}$ [(Pa s)^−1^]
Present study	Porcine	1.2e‐6–4.4e‐5
Di Gregorio et al. [23]	Human	5.5e‐6–1.3e‐4
Michler et al. [19]	Human	2e‐5
Hyde et al. [10]	Porcine	4.49e‐5–2.79e‐3

The model accuracy depends on the number of compartments and the size of the REV. Specifically, three compartments were found to be sufficient for both test cases, while the optimal REV radius was $r_{R}=5\;\mathrm{mm}$ for the geometry of the cube and $r_{R}=2\;\mathrm{mm}$ for the case of left ventricular perfusion (LV). These values provided the closest agreement with the reference pressure distributions from discrete simulations. It is important to note that the optimal compartment configuration and REV size are influenced by factors such as the anatomical scale of the domain, the distribution of vessel radii, and the density of the microvascular network. To generalize these choices for patient‐specific applications, a statistical analysis across a cohort of patients would be necessary, potentially linking the REV and compartment sizes to measurable physiological parameters such as heart size, patient weight, or vascular density metrics.

A key simplification made in this study was the use of a steady‐state formulation for validation purposes. While the general model is capable of capturing unsteady flow dynamics, the steady‐state version enabled direct comparison with Poiseuille flow simulations and allowed us to isolate the effects of spatial heterogeneity and vascular coupling in a controlled setting. As noted in Section 2.1, the steady‐state formulation still incorporates key physical features such as elastic deformation of blood vessels and their coupling with the nonlinear solid deformation of the surrounding tissue. This steady‐state analysis provides meaningful insights into pressure‐driven Darcy flow and serves as a robust first step toward full unsteady simulations.

Furthermore, we assumed the patient‐specific porous parameters to be time‐independent by deriving them from steady‐state Poiseuille flow within the vascular network. Such an approach allows us to obtain patient‐specific porous parameters without incurring significant computational complexity, while still capturing anatomical variability and enabling the modeling of patient‐specific pathologies. The underlying justification is that the vascular architecture remains largely unchanged and is not significantly affected by the cyclic deformation of the myocardium. Therefore, porous parameters evaluated at a reference state can serve as a meaningful approximation. As this work represents a first step toward patient‐specific parameterization, the proposed method offers a substantial improvement over conventional homogeneous models.

## 5 Conclusions and Limitations

In this study, we developed a novel mathematical framework for blood perfusion using a poroelastic model with a multicompartment Darcy flow approach. This model incorporates nonlinear skeletal deformation coupled with FSI in large, small, and microvessels, and accounts for heterogeneous and anisotropic parameterization of the poroelastic medium. We demonstrated that, provided the microvascular network is available, porous parameters for patient‐specific cases can be systematically derived from the vascular geometry, offering a path toward clinically relevant simulations. Numerical results from both a simplified benchmark problem in a cubic domain and realistic perfusion simulations in a porcine left ventricle showed that the derived porous parameters are highly spatially heterogeneous. The permeability tensor was strongly anisotropic in compartments dominated by large vessels and tended toward isotropy in the microvascular compartments.

Using an error metric that compares the continuum Darcy pore pressure in the myocardium with the discrete pressure from the vessel network, we evaluated how accurately the proposed multicompartment Darcy flow model captures pressure distribution across vascular scales. It was concluded that heterogeneity in the porous parameters significantly improves pressure prediction and is therefore essential for accurate perfusion modeling. Although permeability anisotropy had a smaller impact, it still enhanced model accuracy. We also found that the number of vascular compartments used in the Darcy model strongly influences multiscale representation, with three compartments providing the optimal balance between complexity and accuracy for both the benchmark and ventricular simulations. Simulations of patient‐specific pathological conditions, such as vessel occlusions, further highlighted the limitations of homogeneous models in capturing spatial perfusion variability. These results reinforce the importance of using spatially heterogeneous parameterizations when modeling diseased states.

While the proposed framework demonstrates strong capabilities for multiscale cardiac perfusion modeling, the following assumptions and simplifications present opportunities for further refinement and future research.

- Limited availability of high‐resolution vascular data: The full capability of the proposed method relies on detailed clinical or synthetic vascular data. However, such data may not always be available in patient‐specific settings. Current research is progressing toward generating patient‐specific vasculature using hybrid approaches that integrate imaging data with mathematical modelling [39, 40]. Advances in both areas are essential to fully exploit the capabilities of the proposed modeling framework. Additionally, to generalize porous parameters for cases lacking detailed vasculature, extensive simulations on patient cohorts combined with statistical analyses will be necessary. In this context, the proposed model provides a robust platform for conducting such studies.
- Steady state simulations: The model currently uses a steady‐state approximation, which is appropriate for validation but does not capture transient phenomena such as wave propagation and cardiac‐cycle‐induced pressure variations. Since the underlying model supports unsteady simulations, future work will incorporate time‐dependent dynamics and physiological boundary conditions corresponding to systolic and diastolic phases to enhance clinical relevance.
- Limited evaluation of patient‐specific conditions: While the model was applied to a case involving large‐vessel occlusion, other patient‐specific pathologies such as regional stiffness variations, diffuse coronary disease, and microvascular dysfunction were not considered. Future studies will investigate how the model performs under such conditions, further supporting its use in clinical decision support.
- Broader applications not yet explored: Although the framework was developed for cardiac perfusion, its structure supports extension to other physiological transport phenomena, such as species mass transport, representing a promising direction for future research.

## Ethics Statement

The authors have nothing to report.

## Conflicts of Interest

The authors declare no conflicts of interest.

## Figures and Tables

**FIGURE A1:**
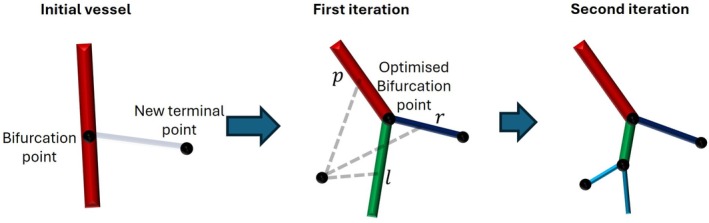
Illustration of vessel network generation using the CCO algorithm. The figure shows an initial vessel segment and the addition of new terminal points selected randomly from a predefined distribution. Each new point is connected to the existing network by forming a bifurcation, where the bifurcation location is optimized to minimize the total vessel volume. The bifurcation consists of a parent vessel p and two daughter vessels l and r. Vessel radii are updated based on Murray's law, and the flow is governed by Poiseuille's law along with flow balance at the junctions. In subsequent iterations, new terminal points are evaluated for possible connection to all existing vessels, and the configuration yielding the minimum total vessel volume is selected. This iterative process continues to construct an optimized, bifurcating vascular tree.

**FIGURE B2:**
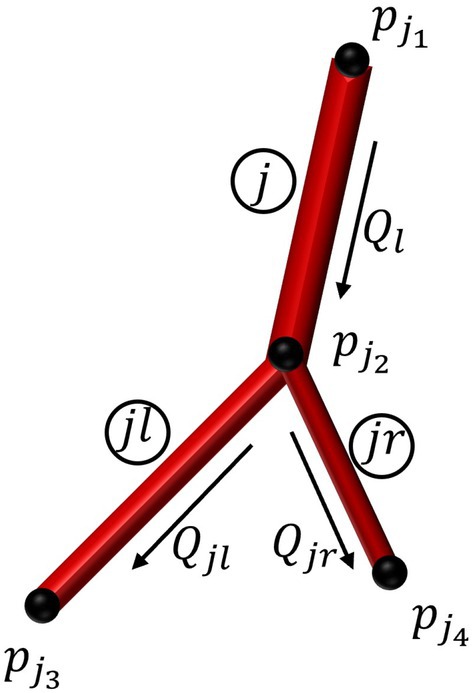
A bifurcating vessel segment illustrating the nodal pressures and mean flow rates used for solving Poiseuille flow. The parent vessel j bifurcates into daughter vessels jl and jr. Nodal pressures are defined at the inlet node j1, the bifurcation junction node j2, and the outlet nodes of the daughter vessels, j3 and j4. Mean flow rates $Q_{j}$, $Q_{\mathrm{jl}}$, and $Q_{\mathrm{jr}}$ are assigned along vessels j, jl, and jr, respectively.

## Data Availability

The data that support the findings of this study are available from the corresponding author upon reasonable request.

## Appendix A Generation of the Vascular Network Using the CCO Algorithm

The blood vessel network within the cube (Figure 1) is generated using the CCO algorithm, following the approach described by Karch et al. [2]. While the detailed implementation is available in the original work, we briefly summarize the model and the steps used to generate the vascular network inside a cubic domain.

In the CCO method, the vascular tree is modeled as a set of bifurcating vessels constructed based on physiological principles. At each bifurcation, the radii of the daughter vessels are related to the radius of the parent vessel through Murray's law,

(A1)
$$r_{p}^{\gamma }=r_{l}^{\gamma }+r_{r}^{\gamma }\;\text{with}\;\gamma =3$$

where $r_{p}$, $r_{l}$, and $r_{r}$ represent the radius of the parent, left daughter, and right daughter vessels, respectively. Flow through each vessel is assumed to be steady and governed by Poiseuille's law,

(A2)
$$Q=\frac{\Delta p}{R}\;\text{with}\;R=\frac{8\mathrm{\mu l}}{\pi r^{4}}$$

where Q is the flow rate, Δp is the pressure drop, R is the hydraulic resistance, μ is the dynamic viscosity, l is the vessel length, and r is the vessel radius.

Flow conservation is enforced at each bifurcation using the balance equation.

(A3)
$$Q_{p}=Q_{l}+Q_{r}$$

where $Q_{p}$ is the flow in the parent vessel, and $Q_{l}$ and $Q_{r}$ are the flows in the left and right daughter vessels, respectively. Additionally, the terminal vessels are assumed to have the same pressure and equally distributed flow.

The terminal points are distributed uniformly within the cube domain. To achieve this, the cube is first discretized using finite elements. Gaussian quadrature points within these elements are then used as potential terminal locations. Initially, a single root vessel of radius $r_{\text{in}}=3\;\mathrm{mm}$ is defined, connecting a distal point at $\left(0,0,50\right)$ to a proximal point at $\left(0,0,-50\right)$. Terminal points are selected using a pseudorandom number generator from the uniform set. In the first iteration, the chosen point is connected to the initial vessel to form a bifurcation (Figure A1). The radii of the bifurcating vessels are updated using Murray's law, and the bifurcation point is repositioned to minimize the total vessel volume. This also minimizes the network's overall hydraulic resistance.

From the second iteration onward, each new terminal point can connect to any existing vessel segment (Figure A1). Among all possible configurations, the connection that results in the minimum total vessel volume is chosen. This iterative optimization ensures the resulting vascular network is both efficient and physiologically realistic.

## Appendix B Poiseuille Flow in the Vessels

The vessel network is modeled as a series of bifurcating vessels. For steady‐state flow through the vascular network, each vessel j (Figure B2) is characterized by nodal pressures $p_{j1}$ and $p_{j2}$ and the mean flow rate $Q_{j}$. These quantities are related by Poiseuille's law,

(B4)
$$Q_{j}=\frac{p_{j1}-p_{j2}}{R_{j}}\;\text{where}\;R_{j}=\frac{\pi r_{j}^{4}}{8\mu l_{j}}$$

At the bifurcation point distal to the vessel (Figure B2), flow conservation is imposed by

(B5)
$$Q_{j}=Q_{\mathrm{jl}}+Q_{\mathrm{jr}}$$

where $Q_{\mathrm{jl}}$ and $Q_{\mathrm{jr}}$ denote the flow rates in the left and right daughter vessels branching from vessel j. Substituting Poiseuille's law (B4) into the flow balance Equation (B5), we obtain the following relationship among the nodal pressures

(B6)
$$\frac{p_{j1}}{R_{j}}+\frac{p_{j3}}{R_{\mathrm{jl}}}+\frac{p_{j4}}{R_{\mathrm{jr}}}-p_{j2}\left(\frac{1}{R_{j}}+\frac{1}{R_{\mathrm{jl}}}+\frac{1}{R_{\mathrm{jr}}}\right)=0$$

where $p_{j3}$ and $p_{j4}$ are the pressures at the distal nodes of the daughter vessels (Figure B2).

The following boundary conditions are applied at the inlet and outlet vessels.

(B7)
$$\begin{array}{r} p_{j1}=p_{\text{in}}\;\left(\text{inlet vessel}\right),\\ p_{j2}=p_{\text{out}}\;\left(\text{outlet vessels}\right) \end{array}$$

where $p_{\text{in}}$ and $p_{\text{out}}$ are the prescribed inlet and outlet pressures of the vascular network. The system of equations governing all bifurcation junctions (B6), along with the boundary Conditions (B7), is solved to determine the nodal pressures throughout the vascular network. Once these pressures are known, the mean Poiseuille pressure and the corresponding mean flow rate for each vessel j in the compartment i are computed as

(B8)
$$p_{j}^{\mathrm{PL}}=\frac{p_{j1}+p_{j2}}{2},\;Q_{j}^{\mathrm{PL}}=Q_{j}=\frac{p_{j1}-p_{j2}}{R_{j}}$$
